# Complete Genome Sequence of the Kiwifruit Pathogen *Pseudomonas syringae* pv. actinidiae Biovar 5, Originating from Japan

**DOI:** 10.1128/genomeA.01409-17

**Published:** 2017-12-14

**Authors:** Russell Poulter, George Taiaroa, Nicholas Sumpter, Peter Stockwell, Margi Butler

**Affiliations:** a Department of Biochemistry, University of Otago, Dunedin, New Zealand; b Department of Microbiology, University of Otago, Dunedin, New Zealand

## Abstract

We present the first complete genome sequence of a copper-resistant biovar 5 strain of a bacterial pathogen of kiwifruit, *Pseudomonas syringae* pv. actinidiae. Comparison with the genome sequence of a copper-sensitive biovar 5 isolate indicates that copper resistance is encoded on a plasmid.

## GENOME ANNOUNCEMENT

*Pseudomonas syringae* pv. actinidiae causes bacterial canker of kiwifruit, a disease that is devastating kiwifruit orchards throughout the world (1). The only effective method of control is to use antibacterial copper compounds. Therefore, the emergence of *P. syringae* pv. actinidiae strains that are resistant to copper poses a major threat to kiwifruit production. Strains of *P. syringae* pv. actinidiae are separated into biovars; the first biovar 5 strains were found in Japan in 2010 (2, 3). We have detected copper-resistant isolates of biovar 5. To determine the basis of copper resistance, we sequenced and assembled the genome of a copper-resistant biovar 5 isolate, MAFF212063, using the PacBio RSII system. The average length of the 146,443 reads was 8,395 bases, with an average depth of coverage of >150×. The reads were assembled using RS_HGAP Assembly.3. The initial assembly was error corrected using 1.89 million Illumina sequence reads. MAFF212063 has a chromosome of 6,556,999 bp and two plasmids of 68,316 bp (pMAFF212063-A) and 68,155 bp (pMAFF212063-B). An integrative conjugative element of 99,148 bp is present between positions 4,717,465 and 4,816,696 (Pac_ICE16). Illumina sequencing of a copper-sensitive isolate of biovar 5 (MAFF212054) was performed for comparison to clarify the basis of copper resistance. The chromosomes of MAFF212063 and MAFF212054 differ at 11 positions, none with a connection to copper resistance. MAFF212054 has only one plasmid, which differs from pMAFF212063-B at a single position.

Copper resistance is therefore encoded on plasmid pMAFF212063-A. This element carries a copper resistance operon encoding CopA, CopB, CopC, CopD, and the regulatory CopRS pair. Nakajima et al. (4) described a very similar plasmid, pPaCu1, from a copper-resistant biovar 1 *P. syringae* pv. actinidiae strain (Pa429). Four pPaCu1 clones present in GenBank (representing 14,819 bp of pPaCu1) are highly similar (99.9% identity) to pMAFF212063-A. There are also highly similar plasmids (differing at only 5 single nucleotide polymorphisms [SNPs]) in copper-resistant *P. syringae* pv. syringae from mango buds in Spain (pPs0081) and from sweet cherry in the United States (pPs6-9) (5). These plasmids do not have transposon Tn*5393* carrying streptomycin resistance genes, which is present in pMAFF212063-A. The level of sequence identity between the copper resistance biovar 5 plasmid and these related plasmids suggests recent horizontal transfer between different pseudomonads. Interestingly, an integrating conjugative element (ICE) (Pac_ICE12, GenBank accession number KY287802) from a New Zealand isolate of *P. syringae* pv. actinidiae biovar 3 is identical to pMAFF212063-A over a >20-kb region that includes the copper resistance genes. This indicates that the copper resistance determinant can occur on diverse mobile elements.

Analysis of the methylation patterns identified by the PacBio analysis showed that strain MAFF212063 has unique restriction systems, a type I GGYANNNNNNTTTC site (99.4% methylation), a type II site CTCGAG (75.7%), and two type III sites recognizing CCTTNAG (98.3%) and GAAGAT (96.2%). Given that the copper resistance plasmids have been transferred between strains of *Pseudomonas syringae*, there must be a method of mitigating the effects of the various restriction systems.

### Accession number(s).

The genome sequences described here have been deposited in DDBJ/ENA/GenBank under the accession numbers CP024712 (MAFF212063 chromosome), CP024713 (plasmid pMAFF212063-A), and CP024714 (plasmid pMAFF212063-B). The whole-genome sequencing (WGS) project for MAFF212054 has been deposited under the GenBank accession number PESZ00000000.
